# Asymptotic Rate-Distortion Analysis of Symmetric Remote Gaussian Source Coding: Centralized Encoding vs. Distributed Encoding

**DOI:** 10.3390/e21020213

**Published:** 2019-02-23

**Authors:** Yizhong Wang, Li Xie, Siyao Zhou, Mengzhen Wang, Jun Chen

**Affiliations:** 1 College of Electronic Information and Automation, Tianjin University of Science and Technology, Tianjin 300222, China; 2 Department of Electrical System of Launch Vehicle, Institute of Aerospace System Engineering Shanghai, Shanghai Academy of Spaceflight Technology, Shanghai 201109, China; 3 Department of Electrical and Computer Engineering, McMaster University, Hamilton, ON L8S 4K1, Canada

**Keywords:** CEO problem, mean squared error, multiterminal source coding, rate-distortion, remote source coding

## Abstract

Consider a symmetric multivariate Gaussian source with *ℓ* components, which are corrupted by independent and identically distributed Gaussian noises; these noisy components are compressed at a certain rate, and the compressed version is leveraged to reconstruct the source subject to a mean squared error distortion constraint. The rate-distortion analysis is performed for two scenarios: centralized encoding (where the noisy source components are jointly compressed) and distributed encoding (where the noisy source components are separately compressed). It is shown, among other things, that the gap between the rate-distortion functions associated with these two scenarios admits a simple characterization in the large *ℓ* limit.

## 1. Introduction

**Figure 1:**
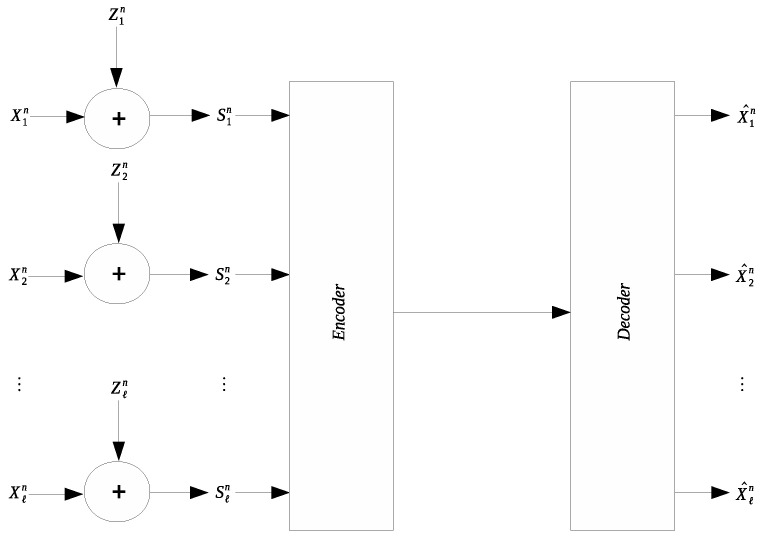
Symmetric remote Gaussian source coding with centralized encoding.

**Figure 2:**
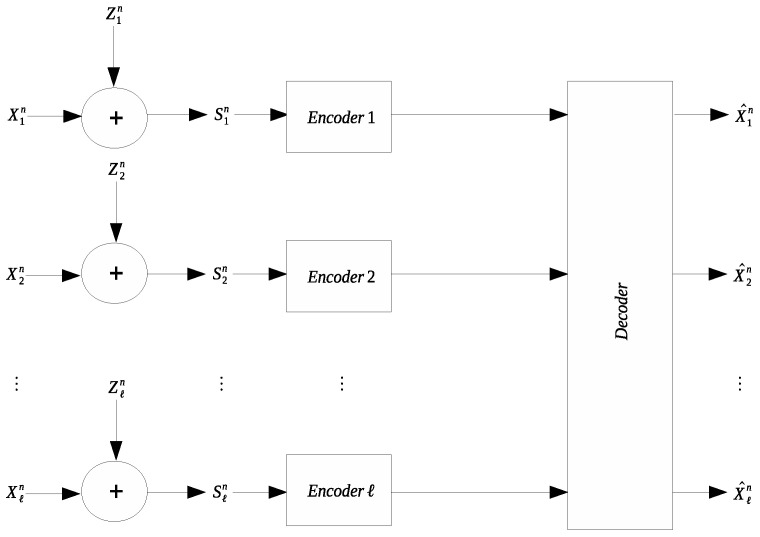
Symmetric remote Gaussian source coding with distributed encoding.

Many applications involve collection and transmission of potentially noise-corrupted data. It is often necessary to compress the collected data to reduce the transmission cost. The remote source coding problem aims to characterize the optimal scheme for such compression and the relevant information-theoretic limit. In this work we study a quadratic Gaussian version of the remote source coding problem, where compression is performed on the noise-corrupted components of a symmetric multivariate Gaussian source. A prescribed mean squared error distortion constraint is imposed on the reconstruction of the noise-free source components; moreover, it is assumed that the noises across different source components are independent and obey the same Gaussian distribution. Two scenarios are considered: centralized encoding (see Figure 1) and distributed encoding (see Figure 2). It is worth noting that the distributed encoding scenario is closely related to the CEO problem, which has been studied extensively [1,2,3,4,5,6,7,8,9,10,11,12,13,14,15,16,17,18].

The present paper is primarily devoted to the comparison of the rate-distortion functions associated with the aforementioned two scenarios. We are particularly interested in understanding how the rate penalty for distributed encoding (relative to centralized encoding) depends on the target distortion as well as the parameters of source and noise models. Although the information-theoretic results needed for this comparison are available in the literature or can be derived in a relatively straightforward manner, the relevant expressions are too unwieldy to analyze. For this reason, we focus on the asymptotic regime where the number of source components, denoted by *ℓ*, is sufficiently large. Indeed, it will be seen that the gap between the two rate-distortion functions admits a simple characterization in the large *ℓ* limit, yielding useful insights into the fundamental difference between centralized encoding and distributed coding, which are hard to obtain otherwise.

The rest of this paper is organized as follows. We state the problem definitions and the main results in Section 2. The proofs are provided in Section 3. We conclude the paper in Section 4.

Notation: The expectation operator and the transpose operator are denoted by E[·] and ${\left(\cdot \right)}^{T}$, respectively. An *ℓ*-dimensional all-one row vector is written as $1_{\ell }$. We use $W^{n}$ as an abbreviation of (W(1),⋯,W(n)). The cardinality of a set C is denoted by |C|. We write g(ℓ)=O(f(ℓ)) if the absolute value of $\frac{g(\ell )}{f(\ell )}$ is bounded for all sufficiently large *ℓ*. Throughout this paper, the base of the logarithm function is *e*, and $\log^{+}x≜\max\left\{\log x,0\right\}$.

## 2. Problem Definitions and Main Results

Let $S≜{\left(S_{1},\cdots ,S_{\ell }\right)}^{T}$ be the sum of two mutually independent *ℓ*-dimensional (ℓ≥2) zero-mean Gaussian random vectors, source $X≜{\left(X_{1},\cdots ,X_{\ell }\right)}^{T}$ and noise $Z≜{\left(Z_{1},\cdots ,Z_{\ell }\right)}^{T}$, with

$$\begin{array}{c} E\left[X_{i}X_{j}\right]=\left\{\begin{array}{cc} \gamma_{X}, & i=j,\\ \rho_{X}\gamma_{X}, & i\neq j, \end{array}\right.\\ E\left[Z_{i}Z_{j}\right]=\left\{\begin{array}{cc} \gamma_{Z}, & i=j,\\ 0, & i\neq j, \end{array}\right. \end{array}$$

where $\gamma_{X}>0$, $\rho_{X}\in \left[\frac{1}{\ell -1},1\right]$, and $\gamma_{Z}\geq 0$. Moreover, let ${\left\{\left(X\left(t\right),Z\left(t\right),S\left(t\right)\right)\right\}}_{t=1}^{\infty }$ be i.i.d. copies of (X,Z,S).

**Definition 1 (**Centralized encoding**).** *A rate-distortion pair (r,d) is said to be achievable with centralized encoding if, for any ϵ>0, there exists an encoding function $\phi^{\left(n\right)}:R^{\ell \times n}\to C^{\left(n\right)}$ such that*

$$\begin{array}{c} \frac{1}{n}\log\left|C^{\left(n\right)}\right|\leq r+\epsilon ,\\ \frac{1}{\ell n}\sum_{i=1}^{\ell }\sum_{t=1}^{n}E\left[{\left(X_{i}\left(t\right)-{\hat{X}}_{i}\left(t\right)\right)}^{2}\right]\leq d+\epsilon , \end{array}$$

*where ${\hat{X}}_{i}\left(t\right)≜E\left[X_{i}\left(t\right)\right|\left(\phi^{\left(n\right)}\left(S^{n}\right))\right]$. For a given d, we denote by $\underline{r}\left(d\right)$ the minimum r such that (r,d) is achievable with centralized encoding*.

**Definition 2 (**Distributed encoding**).** *A rate-distortion pair (r,d) is said to be achievable with distributed encoding if, for any ϵ>0, there exist encoding functions $\phi_{i}^{\left(n\right)}:R^{n}\to C_{i}^{\left(n\right)}$, i=1,⋯,ℓ, such that*

$$\begin{array}{c} \frac{1}{n}\sum_{i=1}^{\ell }\log\left|C_{i}^{\left(n\right)}\right|\leq r+\epsilon ,\\ \frac{1}{\ell n}\sum_{i=1}^{\ell }\sum_{t=1}^{n}E\left[{\left(X_{i}\left(t\right)-{\hat{X}}_{i}\left(t\right)\right)}^{2}\right]\leq d+\epsilon , \end{array}$$

*where ${\hat{X}}_{i}\left(t\right)≜E\left[X_{i}\left(t\right)|\left(\phi_{1}^{\left(n\right)}\left(S_{1}^{n}\right),\cdots ,\phi_{\ell }^{\left(n\right)}\left(S_{\ell }^{n}\right)\right)\right]$. For a given d, we denote by $\bar{r}\left(d\right)$ the minimum r such that (r,d) is achievable with distributed encoding*.

We will refer to $\underline{r}\left(d\right)$ as the rate-distortion function of symmetric remote Gaussian source coding with centralized encoding, and $\bar{r}\left(d\right)$ as the rate-distortion function of symmetric remote Gaussian source coding with distributed encoding. It is clear that $\underline{r}\left(d\right)\leq \bar{r}\left(d\right)$ for any *d* since distributed encoding can be simulated by centralized encoding. Moreover, it is easy to show that $\underline{r}\left(d\right)=\bar{r}\left(d\right)=0$ for $d\geq \gamma_{X}$ (since the distortion constraint is trivially satisfied with the reconstruction set to be zero) and $\underline{r}\left(d\right)=\bar{r}\left(d\right)=\infty$ for $d\leq d_{\min}$ (since $d_{\min}$ is the minimum achievable distortion when ${\left\{S\left(t\right)\right\}}_{t=1}^{\infty }$ is directly available at the decoder), where (see Section 3.1 for a detailed derivation)

$$\begin{array}{c} d_{\min}≜\frac{1}{\ell }E\left[{\left(X-E\left[X|S\right]\right)}^{T}\left(X-E\left[X|S\right]\right)\right]=\left\{\begin{array}{cc} \frac{\left(\ell -1\right)\gamma_{X}\gamma_{Z}}{\ell \gamma_{X}+\left(\ell -1\right)\gamma_{Z}}, & \rho_{X}=-\frac{1}{\ell -1},\\ \frac{\left(\ell \rho_{X}\gamma_{X}+\lambda_{X}\right)\gamma_{Z}}{\ell (\ell \rho_{X}\gamma_{X}+\lambda_{X}+\gamma_{Z})}+\frac{\left(\ell -1\right)\lambda_{X}\gamma_{Z}}{\ell (\lambda_{X}+\gamma_{Z})}, & \rho_{X}\in \left(-\frac{1}{\ell -1},1\right),\\ \frac{\gamma_{X}\gamma_{Z}}{\ell \gamma_{X}+\gamma_{Z}}, & \rho_{X}=1, \end{array}\right. \end{array}$$

with $\lambda_{X}≜\left(1-\rho_{X}\right)\gamma_{X}$. Henceforth we shall focus on the case $d\in (d_{\min},\gamma_{X})$.

**Lemma** **1.** *For $d\in (d_{\min},\gamma_{X})$,*

$$\begin{array}{c} \underline{r}\left(d\right)=\left\{\begin{array}{cc} \frac{\ell -1}{2}\log\frac{\ell \left(\ell -1\right)\gamma_{X}^{2}}{\left(\ell \gamma_{X}+\left(\ell -1\right)\gamma_{Z}\right)\left(\left(\ell -1\right)d-\gamma_{X}\right)}, & \rho_{X}=-\frac{1}{\ell -1},\\ \frac{1}{2}\log^{+}\frac{{\left(\ell \rho_{X}\gamma_{X}+\lambda_{X}\right)}^{2}}{(\ell \rho_{X}\gamma_{X}+\lambda_{X}+\gamma_{Z})\xi }+\frac{\ell -1}{2}\log^{+}\frac{\lambda_{X}^{2}}{(\lambda_{X}+\gamma_{Z})\xi }, & \rho_{X}\in \left(-\frac{1}{\ell -1},1\right),\\ \frac{1}{2}\log\frac{\ell \gamma_{X}^{2}}{\left(\ell \gamma_{X}+\gamma_{Z}\right)d-\gamma_{X}\gamma_{Z}}, & \rho_{X}=1, \end{array}\right. \end{array}$$

*where*

$$\begin{array}{c} \xi ≜\left\{\begin{array}{cc} d-d_{\min}, & d\leq \min\left\{\frac{{\left(\ell \rho_{X}\gamma_{X}+\lambda_{X}\right)}^{2}}{\ell \rho_{X}\gamma_{X}+\lambda_{X}+\gamma_{Z}},\frac{\lambda_{X}^{2}}{\lambda_{X}+\gamma_{Z}}\right\}+d_{\min},\\ \frac{\ell (d-d_{\min})}{\ell -1}-\frac{{\left(\ell \rho_{X}\gamma_{X}+\lambda_{X}\right)}^{2}}{\left(\ell -1\right)\left(\ell \rho_{X}\gamma_{X}+\lambda_{X}+\gamma_{Z}\right)}, & d>\frac{{\left(\ell \rho_{X}\gamma_{X}+\lambda_{X}\right)}^{2}}{\ell \rho_{X}\gamma_{X}+\lambda_{X}+\gamma_{Z}}+d_{\min},\\ \ell \left(d-d_{\min}\right)-\frac{\left(\ell -1\right)\lambda_{X}^{2}}{\lambda_{X}+\gamma_{Z}}, & d>\frac{\lambda_{X}^{2}}{\lambda_{X}+\gamma_{Z}}+d_{\min}. \end{array}\right. \end{array}$$

**Proof.**  See Section 3.1. □

The following result can be deduced from ([19] Theorem 1) (see also [11,15]).

**Lemma** **2.** *For $d\in (d_{\min},\gamma_{X})$,*

$$\begin{array}{c} \bar{r}\left(d\right)=\frac{1}{2}\log\frac{\ell \rho_{X}\gamma_{X}+\lambda_{X}+\gamma_{Z}+\lambda_{Q}}{\lambda_{Q}}+\frac{\ell -1}{2}\log\frac{\lambda_{X}+\gamma_{Z}+\lambda_{Q}}{\lambda_{Q}}, \end{array}$$

*where*

$$\begin{array}{c} \lambda_{Q}≜\frac{-b+\sqrt{b^{2}-4ac}}{2a} \end{array}$$

*with*

$$\begin{array}{cc} a & ≜\ell (\gamma_{X}-d),\\ b & ≜\left(\ell \rho_{X}\gamma_{X}+\lambda_{X}\right)\left(\lambda_{X}+2\gamma_{Z}\right)+\left(\ell -1\right)\lambda_{X}\left(\ell \rho_{X}\gamma_{X}+\lambda_{X}+2\gamma_{Z}\right)-\ell \left(\ell \rho_{X}\gamma_{X}+2\lambda_{X}+2\gamma_{Z}\right)d,\\ c & ≜\ell \left(\ell \rho_{X}\gamma_{X}+\lambda_{X}+\gamma_{Z}\right)\left(\lambda_{X}+\gamma_{Z}\right)\left(d_{\min}-d\right). \end{array}$$

The expressions of $\underline{r}\left(d\right)$ and $\bar{r}\left(d\right)$ as shown in Lemmas 1 and 2 are quite complicated, rendering it difficult to make analytical comparisons. Fortunately, they become significantly simplified in the asymptotic regime where ℓ→∞ (with *d* fixed). To perform this asymptotic analysis, it is necessary to restrict attention to the case $\rho_{X}\in \left[0,1\right]$; moreover, without loss of generality, we assume $d\in (d_{\min}^{\left(\infty \right)},\gamma_{X})$, where

$$\begin{array}{c} d_{\min}^{\left(\infty \right)}≜\lim_{\ell \to \infty }d_{\min}=\left\{\begin{array}{cc} \frac{\lambda_{X}\gamma_{Z}}{\lambda_{X}+\gamma_{Z}}, & \rho_{X}\in \left[0,1\right),\\ 0, & \rho_{X}=1. \end{array}\right. \end{array}$$

**Theorem 1 (**Centralized encoding**).**

*1.*  *$\rho_{X}=0$: For $d\in (d_{\min}^{\left(\infty \right)},\gamma_{X})$,*
  $$\begin{array}{c} \underline{r}\left(d\right)=\frac{\ell }{2}\log\frac{\gamma_{X}^{2}}{\left(\gamma_{X}+\gamma_{Z}\right)d-\gamma_{X}\gamma_{Z}}. \end{array}$$
*2.*  *$\rho_{X}\in \left(0,1\right]$: For $d\in (d_{\min}^{\left(\infty \right)},\gamma_{X})$,*
  $$\begin{array}{c} \underline{r}\left(d\right)=\left\{\begin{array}{cc} \frac{\ell }{2}\log\frac{\lambda_{X}^{2}}{\left(\lambda_{X}+\gamma_{Z}\right)d-\lambda_{X}\gamma_{Z}}+\frac{1}{2}\log\ell +\underline{\alpha }+O\left(\frac{1}{\ell }\right), & d<\lambda_{X},\\ \frac{1}{2}\log\ell +\frac{1}{2}\log\frac{\rho_{X}\gamma_{X}\left(\lambda_{X}+\gamma_{Z}\right)}{\lambda_{X}^{2}}+\frac{\gamma_{Z}^{2}}{2\lambda_{X}^{2}}+O\left(\frac{1}{\ell }\right), & d=\lambda_{X},\\ \frac{1}{2}\log\frac{\rho_{X}\gamma_{X}}{d-\lambda_{X}}+O\left(\frac{1}{\ell }\right), & d>\lambda_{X}, \end{array}\right. \end{array}$$
  *where*
  $$\begin{array}{c} \underline{\alpha }≜\frac{1}{2}\log\frac{\rho_{X}\gamma_{X}\left(\lambda_{X}+\gamma_{Z}\right)}{\lambda_{X}^{2}}+\frac{\gamma_{Z}^{2}}{2(\left(\lambda_{X}+\gamma_{Z}\right)d-\lambda_{X}\gamma_{Z})}. \end{array}$$

**Proof.**  See Section 3.2. □

**Theorem 2 (**Distributed encoding**).**

*1.*  *$\rho_{X}=0$: For $d\in (d_{\min}^{\left(\infty \right)},\gamma_{X})$,*
  $$\begin{array}{c} \bar{r}\left(d\right)=\frac{\ell }{2}\log\frac{\gamma_{X}^{2}}{\left(\gamma_{X}+\gamma_{Z}\right)d-\gamma_{X}\gamma_{Z}}. \end{array}$$
*2.*  *$\rho_{X}\in \left(0,1\right]$: For $d\in (d_{\min}^{\left(\infty \right)},\gamma_{X})$,*
  $$\begin{array}{c} \bar{r}\left(d\right)=\left\{\begin{array}{cc} \frac{\ell }{2}\log\frac{\lambda_{X}^{2}}{\left(\lambda_{X}+\gamma_{Z}\right)d-\lambda_{X}\gamma_{Z}}+\frac{1}{2}\log\ell +\bar{\alpha }+O\left(\frac{1}{\ell }\right), & d<\lambda_{X},\\ \frac{\left(\lambda_{X}+\gamma_{Z}\right)\sqrt{\ell }}{2\lambda_{X}}+\frac{1}{4}\log\ell +\frac{1}{2}\log\frac{\rho_{X}}{1-\rho_{X}}-\frac{\left(\lambda_{X}+\gamma_{Z}\right)\left(\lambda_{X}-\rho_{X}\gamma_{Z}\right)}{4\rho_{X}\lambda_{X}^{2}}+O\left(\frac{1}{\sqrt{\ell }}\right), & d=\lambda_{X},\\ \frac{1}{2}\log\frac{\rho_{X}\gamma_{X}}{d-\lambda_{X}}+\frac{\left(\lambda_{X}+\gamma_{Z}\right)\left(\gamma_{X}-d\right)}{2\rho_{X}\gamma_{X}\left(d-\lambda_{X}\right)}+O\left(\frac{1}{\ell }\right), & d>\lambda_{X}, \end{array}\right. \end{array}$$
  *where*
  $$\begin{array}{c} \bar{\alpha }≜\frac{1}{2}\log\frac{\rho_{X}\gamma_{X}\left(\lambda_{X}-d\right)}{\lambda_{X}^{2}}+\frac{\left(\lambda_{X}+\gamma_{Z}\right)d^{2}}{2\left(\lambda_{X}-d\right)\left(\left(\lambda_{X}+\gamma_{Z}\right)d-\lambda_{X}\gamma_{Z}\right)}. \end{array}$$

**Proof.**  See Section 3.3. □

**Remark** **1.** *One can readily recover ([20] Theorem 3) for the case m=1 (see [20] for the definition of parameter m) and Oohama’s celebrated result for the quadratic Gaussian CEO problem ([3] Corollary 1) by setting $\gamma_{Z}=0$ and $\rho_{X}=1$, respectively, in Theorem 2*.

The following result is a simple corollary of Theorems 1 and 2.

**Corollary 1 (**Asymptotic gap**).**

*1.*  *$\rho_{X}=0$: For $d\in (d_{\min}^{\left(\infty \right)},\gamma_{X})$,*
  $$\begin{array}{c} \bar{r}\left(d\right)-\underline{r}\left(d\right)=0. \end{array}$$
*2.*  *$\rho_{X}\in \left(0,1\right]$: For $d\in (d_{\min}^{\left(\infty \right)},\gamma_{X})$,*
  $$\begin{array}{c} \lim_{\ell \to \infty }\bar{r}\left(d\right)-\underline{r}\left(d\right)=\psi \left(d\right)≜\left\{\begin{array}{cc} \frac{1}{2}\log\frac{\lambda_{X}-d}{\lambda_{X}+\gamma_{Z}}+\frac{\gamma_{Z}+d}{2(\lambda_{X}-d)}, & d<\lambda_{X},\\ \infty , & d=\lambda_{X},\\ \frac{\left(\lambda_{X}+\gamma_{Z}\right)\left(\gamma_{X}-d\right)}{2\rho_{X}\gamma_{X}\left(d-\lambda_{X}\right)}, & d>\lambda_{X}. \end{array}\right. \end{array}$$

**Figure 3:**
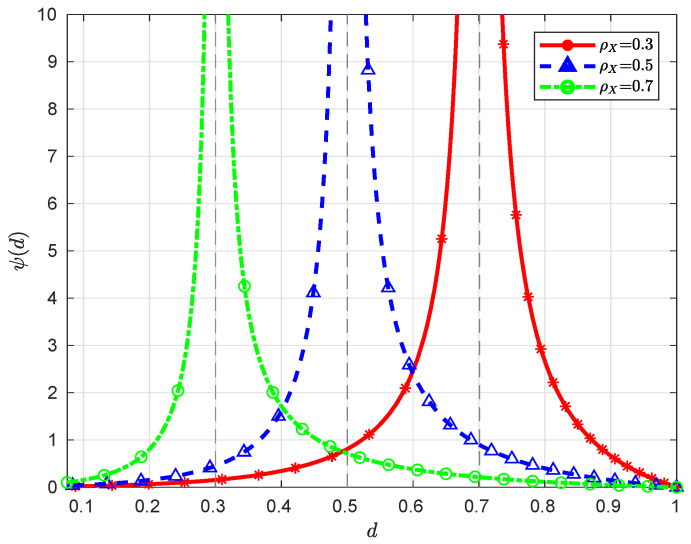
Illustration of ψ(d) with $\gamma_{X}=1$ and $\gamma_{Z}=0.1$ for different $\rho_{X}$.

**Figure 4:**
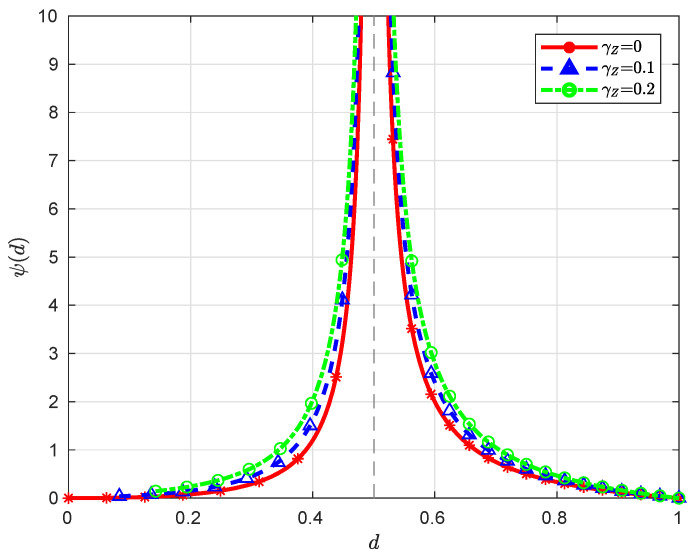
Illustration of ψ(d) with $\gamma_{X}=1$ and $\rho_{X}=0.5$ for different $\gamma_{Z}$.

**Remark** **2.** *When $\rho_{X}=0$, we have $\psi \left(d\right)=\frac{\gamma_{Z}\left(\gamma_{X}-d\right)}{2\gamma_{X}d}$, which is a monotonically decreasing function over $\left(0,\gamma_{X}\right)$, converging to ∞ (here we assume $\gamma_{Z}>0$) and 0 as d→0 and $\gamma_{X}$, respectively. When $\rho_{X}\in \left(0,1\right)$, it is clear that the function ψ(d) is monotonically decreasing over $\left(\lambda_{X},\gamma_{X}\right)$, converging to ∞ and 0 as $d\to \lambda_{X}$ and $\gamma_{X}$, respectively; moreover, since $\psi^{\prime }\left(d\right)=\frac{\gamma_{Z}+d}{2{\left(\lambda_{X}-d\right)}^{2}}>0$ for $d\in (d_{\min}^{\left(\infty \right)},\lambda_{X})$, the function ψ(d) is monotonically increasing over $\left(d_{\min}^{\left(\infty \right)},\lambda_{X}\right)$, converging to $\tau \left(\gamma_{Z}\right)≜\frac{1}{2}\log\frac{\lambda_{X}^{2}}{{\left(\lambda_{X}+\gamma_{Z}\right)}^{2}}+\frac{2\lambda_{X}\gamma_{Z}+\gamma_{Z}^{2}}{2\lambda_{X}^{2}}$ and ∞ as $d\to d_{\min}^{\left(\infty \right)}$ and $\lambda_{X}$, respectively. Note that $\tau^{\prime }\left(\gamma_{Z}\right)=\frac{2\lambda_{X}\gamma_{Z}+\gamma_{Z}^{2}}{\lambda_{X}^{2}\left(\lambda_{X}+\gamma_{Z}\right)}\geq 0$ for $\gamma_{Z}\in \left[0,\infty \right)$; therefore, the minimum value of $\tau (\gamma_{Z})$ over [0,∞) is 0, which is attained at $\gamma_{Z}=0$. See Figure 3 and Figure 4 for some graphical illustrations of ψ(d)*.

## 3. Proofs

### 3.1. Proof of Lemma 1

It is known [21] that $\underline{r}\left(d\right)$ is given by the solution to the following optimization problem:

$$\begin{array}{cc} \left(P_{1}\right) & \min_{p_{\hat{X}|S}}I\left(S;\hat{X}\right)\\ \mathrm{subject}\;\mathrm{to} & E[{\left(X-\hat{X}\right)}^{T}\left(X-\hat{X}\right)]\leq \ell d,\\ & X↔S↔\hat{X}\;\mathrm{form}\;a\;\mathrm{Markov}\;\mathrm{chain}. \end{array}$$

Let $\tilde{X}≜\Theta X$, $\tilde{Z}≜\Theta Z$, and $\tilde{S}≜\Theta S$, where Θ is an arbitrary (real) unitary matrix with the first row being $\frac{1}{\sqrt{\ell }}1_{\ell }$. Since unitary transformations are invertible and preserve the Euclidean norm, we can write $\left(P_{1}\right)$ equivalently as

$$\begin{array}{cc} \left(P_{2}\right) & \min_{p_{\hat{X}|\tilde{S}}}I\left(\tilde{S};\hat{X}\right)\\ \mathrm{subject}\;\mathrm{to} & E[{\left(\tilde{X}-\hat{X}\right)}^{T}\left(\tilde{X}-\hat{X}\right)]\leq \ell d,\\ & \tilde{X}↔\tilde{S}↔\hat{X}\;\mathrm{form}\;a\;\mathrm{Markov}\;\mathrm{chain}. \end{array}$$

For the same reason, we have

(1) $$\begin{array}{c} \ell d_{\min}=E\left[{\left(\tilde{X}-E\left[\tilde{X}|\tilde{S}\right]\right)}^{T}\left(\tilde{X}-E\left[\tilde{X}|\tilde{S}\right]\right)\right]. \end{array}$$

Denote the *i*-th components of $\tilde{X}$, $\tilde{Z}$, and $\tilde{S}$ by ${\tilde{X}}_{i}$, ${\tilde{Z}}_{i}$, and ${\tilde{S}}_{i}$, respectively, i=1,⋯,ℓ. Clearly, ${\tilde{S}}_{i}={\tilde{X}}_{i}+{\tilde{Z}}_{i}$, i=1,⋯,ℓ. Moreover, it can be verified that ${\tilde{X}}_{1},\cdots ,{\tilde{X}}_{\ell },{\tilde{Z}}_{1},\cdots ,{\tilde{Z}}_{\ell }$ are independent zero-mean Gaussian random variables with

(2) $$\begin{array}{c} E\left[{\left({\tilde{X}}_{1}\right)}^{2}\right]=\ell \rho_{X}\gamma_{X}+\lambda_{X}, \end{array}$$

(3) $$\begin{array}{c} E\left[{\left({\tilde{X}}_{i}\right)}^{2}\right]=\lambda_{X},\;i=2,\cdots ,\ell , \end{array}$$

$$\begin{array}{c} E\left[{\left({\tilde{Z}}_{1}\right)}^{2}\right]=\gamma_{Z},\;i=1,\cdots ,\ell . \end{array}$$

Now denote the *i*-th component of $\hat{S}≜E\left[\tilde{X}|\tilde{S}\right]$ by ${\hat{S}}_{i}$, i=1,⋯,ℓ. We have

$$\begin{array}{c} {\hat{S}}_{i}=E\left[{\tilde{X}}_{i}|{\tilde{S}}_{i}\right],\;i=1,\cdots ,\ell , \end{array}$$

and

(4) $$\begin{array}{c} E\left[{\left({\hat{S}}_{1}\right)}^{2}\right]=\left\{\begin{array}{cc} 0, & \rho_{X}=-\frac{1}{\ell -1},\\ \frac{{\left(\ell \rho_{X}\gamma_{X}+\lambda_{X}\right)}^{2}}{\ell \rho_{X}\gamma_{X}+\lambda_{X}+\gamma_{Z}}, & \rho_{X}\in \left(-\frac{1}{\ell -1},1\right], \end{array}\right. \end{array}$$

(5) $$\begin{array}{c} E\left[{\left({\hat{S}}_{i}\right)}^{2}\right]=\left\{\begin{array}{cc} \frac{\lambda_{X}^{2}}{\lambda_{X}+\gamma_{Z}}, & \rho \in [-\frac{1}{\ell -1},1),\\ 0, & \rho_{X}=1, \end{array}\right.\;i=2,\cdots ,\ell . \end{array}$$

Note that

$$\begin{array}{c} E\left[{\left(\tilde{X}-\hat{S}\right)}^{T}\left(\tilde{X}-\hat{S}\right)\right]=\sum_{i=1}^{\ell }E\left[{\left({\tilde{X}}_{i}\right)}^{2}\right]-\sum_{i=1}^{\ell }E\left[{\left({\hat{S}}_{i}\right)}^{2}\right], \end{array}$$

which, together with (1)–(5), proves

$$\begin{array}{c} d_{\min}=\frac{1}{\ell }E\left[{\left(\tilde{X}-\hat{S}\right)}^{T}\left(\tilde{X}-\hat{S}\right)\right]=\left\{\begin{array}{cc} \frac{\left(\ell -1\right)\gamma_{X}\gamma_{Z}}{\ell \gamma_{X}+\left(\ell -1\right)\gamma_{Z}}, & \rho_{X}=-\frac{1}{\ell -1},\\ \frac{\left(\ell \rho_{X}\gamma_{X}+\lambda_{X}\right)\gamma_{Z}}{\ell (\ell \rho_{X}\gamma_{X}+\lambda_{X}+\gamma_{Z})}+\frac{\left(\ell -1\right)\lambda_{X}\gamma_{Z}}{\ell (\lambda_{X}+\gamma_{Z})}, & \rho_{X}\in \left(-\frac{1}{\ell -1},1\right),\\ \frac{\gamma_{X}\gamma_{Z}}{\ell \gamma_{X}+\gamma_{Z}}, & \rho_{X}=1. \end{array}\right. \end{array}$$

Clearly, $\hat{S}$ is determined by $\tilde{S}$; moreover, for any *ℓ*-dimensional random vector $\hat{X}$ jointly distributed with $\left(\tilde{X},\tilde{S}\right)$ such that $\tilde{X}↔\tilde{S}↔\hat{X}$ form a Markov chain, we have

$$\begin{array}{cc} E[{\left(\tilde{X}-\hat{X}\right)}^{T}\left(\tilde{X}-\hat{X}\right)] & =E\left[{\left(\hat{S}-\hat{X}\right)}^{T}{\left(\hat{S}-\hat{X}\right)}^{2}\right]+E\left[{\left(\tilde{X}-\hat{S}\right)}^{T}{\left(\tilde{X}-\hat{S}\right)}^{2}\right]\\ & =E\left[{\left(\hat{S}-\hat{X}\right)}^{T}{\left(\hat{S}-\hat{X}\right)}^{2}\right]+\ell d_{\min}. \end{array}$$

Therefore, $\left(P_{2}\right)$ is equivalent to

$$\begin{array}{cc} \left(P_{3}\right) & \min_{p_{\hat{X}|\hat{S}}}I\left(\hat{S};\hat{X}\right)\\ \mathrm{subject}\;\mathrm{to} & E\left[{\left(\hat{S}-\hat{X}\right)}^{T}\left(\hat{S}-\hat{X}\right)\right]\leq \ell \left(d-d_{\min}\right). \end{array}$$

One can readily complete the proof of Lemma 1 by recognizing that the solution to $\left(P_{3}\right)$ is given by the well-known reverse water-filling formula ([22] Theorem 13.3.3).

### 3.2. Proof of Theorem 1

Setting $\rho_{X}=0$ in Lemma 1 gives

$$\begin{array}{c} \underline{r}\left(d\right)=\frac{\ell }{2}\log\frac{\gamma_{X}^{2}}{\left(\gamma_{X}+\gamma_{Z}\right)d-\gamma_{X}\gamma_{Z}} \end{array}$$

for $d\in (\frac{\gamma_{X}\gamma_{Z}}{\gamma_{X}+\gamma_{Z}},\gamma_{X})$. Setting $\rho_{X}=1$ in Lemma 1 gives

$$\begin{array}{c} \underline{r}\left(d\right)=\frac{1}{2}\log\frac{\ell^{2}\gamma_{X}^{2}}{\ell \left(\ell \gamma_{X}+\gamma_{Z}\right)d-\gamma_{X}\gamma_{Z}} \end{array}$$

for $d\in (\frac{\gamma_{X}\gamma_{Z}}{\ell \gamma_{X}+\gamma_{Z}},\gamma_{X})$; moreover, we have

$$\begin{array}{c} \frac{1}{2}\log\frac{\ell^{2}\gamma_{X}^{2}}{\ell \left(\ell \gamma_{X}+\gamma_{Z}\right)d-\gamma_{X}\gamma_{Z}}=\frac{1}{2}\log\frac{\gamma_{X}}{d}+O\left(\frac{1}{\ell }\right), \end{array}$$

and $\frac{\gamma_{X}\gamma_{Z}}{\ell \gamma_{X}+\gamma_{Z}}\to 0$ as ℓ→∞.

It remains to treat the case $\rho_{X}\in \left(0,1\right)$. In this case, it can be deduced from Lemma 1 that

$$\begin{array}{c} \underline{r}\left(d\right)=\left\{\begin{array}{cc} \frac{1}{2}\log\frac{{\left(\ell \rho_{X}\gamma_{X}+\lambda_{X}\right)}^{2}\left(\lambda_{X}+\gamma_{Z}\right)}{\lambda_{X}^{2}\left(\ell \rho_{X}\gamma_{X}+\lambda_{X}+\gamma_{Z}\right)}+\frac{\ell }{2}\log\frac{\lambda_{X}^{2}}{\left(\lambda_{X}+\gamma_{Z}\right)\left(d-d_{\min}\right)}, & d\in (d_{\min},\frac{\lambda_{X}^{2}}{\lambda_{X}+\gamma_{Z}}+d_{\min}],\\ \frac{1}{2}\log\frac{{\left(\ell \rho_{X}\gamma_{X}+\lambda_{X}\right)}^{2}\left(\lambda_{X}+\gamma_{Z}\right)}{\left(\ell \rho_{X}\gamma_{X}+\lambda_{X}+\gamma_{Z}\right)\left(\ell \left(\lambda_{X}+\gamma_{Z}\right)\left(d-d_{\min}\right)-\left(\ell -1\right)\lambda_{X}^{2}\right)}, & d\in (\frac{\lambda_{X}^{2}}{\lambda_{X}+\gamma_{Z}}+d_{\min},\gamma_{X}), \end{array}\right. \end{array}$$

and we have

(6) $$\begin{array}{cc} d_{\min} & =\frac{\left(\ell \rho_{X}\gamma_{X}+\lambda_{X}\right)\gamma_{Z}}{\ell (\ell \rho_{X}\gamma_{X}+\lambda_{X}+\gamma_{Z})}+\frac{\left(\ell -1\right)\lambda_{X}\gamma_{Z}}{\ell (\lambda_{X}+\gamma_{Z})}\\ & =\frac{\lambda_{X}\gamma_{Z}}{\lambda_{X}+\gamma_{Z}}+\frac{\rho_{X}\gamma_{X}\gamma_{Z}^{2}}{\left(\ell \rho_{X}\gamma_{X}+\lambda_{X}+\gamma_{Z}\right)\left(\lambda_{X}+\gamma_{Z}\right)} \end{array}$$

(7) $$\begin{array}{c} =\frac{\lambda_{X}\gamma_{Z}}{\lambda_{X}+\gamma_{Z}}+\frac{\gamma_{Z}^{2}}{(\lambda_{X}+\gamma_{Z})\ell }+O\left(\frac{1}{\ell^{2}}\right). \end{array}$$

Consider the following two subcases separately.

- $d\in (\frac{\lambda_{X}\gamma_{Z}}{\lambda_{X}+\gamma_{Z}},\lambda_{X}]$
  It can be seen from (6) that $d_{\min}$ is a monotonically decreasing function of *ℓ* and converges to $\frac{\lambda_{X}\gamma_{Z}}{\lambda_{X}+\gamma_{Z}}$ as ℓ→∞. Therefore, we have $d\in (d_{\min},\frac{\lambda_{X}^{2}}{\lambda_{X}+\gamma_{Z}}+d_{\min}]$ and consequently
  (8) $$\begin{array}{c} \underline{r}\left(d\right)=\frac{1}{2}\log\frac{{\left(\ell \rho_{X}\gamma_{X}+\lambda_{X}\right)}^{2}\left(\lambda_{X}+\gamma_{Z}\right)}{\lambda_{X}^{2}\left(\ell \rho_{X}\gamma_{X}+\lambda_{X}+\gamma_{Z}\right)}+\frac{\ell }{2}\log\frac{\lambda_{X}^{2}}{\left(\lambda_{X}+\gamma_{Z}\right)\left(d-d_{\min}\right)}, \end{array}$$
  when *ℓ* is sufficiently large. Note that
  (9) $$\begin{array}{c} \frac{1}{2}\log\frac{{\left(\ell \rho_{X}\gamma_{X}+\lambda_{X}\right)}^{2}}{\ell \rho_{X}\gamma_{X}+\lambda_{X}+\gamma_{Z}}=\frac{1}{2}\log\ell +\frac{1}{2}\log\left(\rho_{X}\gamma_{X}\right)+O\left(\frac{1}{\ell }\right) \end{array}$$
  and
  (10) $$\begin{array}{cc} \frac{1}{2}\log\left(d-d_{\min}\right) & =\frac{1}{2}\log\left(d-\frac{\lambda_{X}\gamma_{Z}}{\lambda_{X}+\gamma_{Z}}-\frac{\gamma_{Z}^{2}}{(\lambda_{X}+\gamma_{Z})\ell }-O\left(\frac{1}{\ell^{2}}\right)\right) \end{array}$$
  (11) $$\begin{array}{c} =\frac{1}{2}\log\frac{\left(\lambda_{X}+\gamma_{Z}\right)d-\lambda_{X}\gamma_{Z}}{\lambda_{X}+\gamma_{Z}}-\frac{\gamma_{Z}^{2}}{2(\left(\lambda_{X}+\gamma_{Z}\right)d-\lambda_{X}\gamma_{Z})\ell }+O\left(\frac{1}{\ell^{2}}\right), \end{array}$$
  where (10) is due to (7). Substituting (9) and (11) into (8) gives
  $$\begin{array}{cc} \underline{r}\left(d\right) & =\frac{\ell }{2}\log\frac{\lambda_{X}^{2}}{\left(\lambda_{X}+\gamma_{Z}\right)d-\lambda_{X}\gamma_{Z}}+\frac{1}{2}\log\ell +\frac{1}{2}\log\frac{\rho_{X}\gamma_{X}\left(\lambda_{X}+\gamma_{Z}\right)}{\lambda_{X}^{2}}\\ & \;+\frac{\gamma_{Z}^{2}}{2(\left(\lambda_{X}+\gamma_{Z}\right)d-\lambda_{X}\gamma_{Z})}+O\left(\frac{1}{\ell }\right). \end{array}$$
  In particular, we have
  $$\begin{array}{c} \underline{r}\left(\lambda_{X}\right)=\frac{1}{2}\log\ell +\frac{1}{2}\log\frac{\rho_{X}\gamma_{X}\left(\lambda_{X}+\gamma_{Z}\right)}{\lambda_{X}^{2}}+\frac{\gamma_{Z}^{2}}{2\lambda_{X}^{2}}+O\left(\frac{1}{\ell }\right). \end{array}$$
- $d\in (\lambda_{X},\gamma_{X})$
  Since $d_{\min}$ converges to $\frac{\lambda_{X}\gamma_{Z}}{\lambda_{X}+\gamma_{Z}}$ as ℓ→∞, it follows that $d\in (\frac{\lambda_{X}^{2}}{\lambda_{X}+\gamma_{Z}}+d_{\min},\gamma_{X})$ and consequently
  (12) $$\begin{array}{c} \underline{r}\left(d\right)=\frac{1}{2}\log\frac{{\left(\ell \rho_{X}\gamma_{X}+\lambda_{X}\right)}^{2}\left(\lambda_{X}+\gamma_{Z}\right)}{\left(\ell \rho_{X}\gamma_{X}+\lambda_{X}+\gamma_{Z}\right)\left(\ell \left(\lambda_{X}+\gamma_{Z}\right)\left(d-d_{\min}\right)-\left(\ell -1\right)\lambda_{X}^{2}\right)} \end{array}$$
  when *ℓ* is sufficiently large. One can readily verify that
  (13) $$\begin{array}{c} \frac{1}{2}\log\frac{{\left(\ell \rho_{X}\gamma_{X}+\lambda_{X}\right)}^{2}}{\left(\ell \rho_{X}\gamma_{X}+\lambda_{X}+\gamma_{Z}\right)\left(\ell \left(\lambda_{X}+\gamma_{Z}\right)\left(d-d_{\min}\right)-\left(\ell -1\right)\lambda_{X}^{2}\right)}\\ =\frac{1}{2}\log\frac{\rho_{X}\gamma_{X}}{\left(\lambda_{X}+\gamma_{Z}\right)\left(d-\lambda_{X}\right)}+O\left(\frac{1}{\ell }\right). \end{array}$$
  Substituting (13) into (12) gives
  $$\begin{array}{c} \underline{r}\left(d\right)=\frac{1}{2}\log\frac{\rho_{X}\gamma_{X}}{d-\lambda_{X}}+O\left(\frac{1}{\ell }\right). \end{array}$$
  This completes the proof of Theorem 1.

### 3.3. Proof of Theorem 2

One can readily prove part one of Theorem 2 by setting $\rho_{X}=0$ in Lemma 2. So only part two of Theorem 2 remains to be proved. Note that

$$\begin{array}{c} b=g_{1}\ell^{2}+g_{2}\ell ,\\ c=h_{1}\ell^{2}+h_{2}\ell , \end{array}$$

where

$$\begin{array}{c} g_{1}≜\rho_{X}\gamma_{X}\left(\lambda_{X}-d\right),\\ g_{2}≜\lambda_{X}^{2}+2\gamma_{X}\gamma_{Z}-2\left(\lambda_{X}+\gamma_{Z}\right)d,\\ h_{1}≜\rho_{X}\gamma_{X}\left(\lambda_{X}+\gamma_{Z}\right)\left(d_{\min}^{\left(\infty \right)}-d\right),\\ h_{2}≜\rho_{X}\gamma_{X}\gamma_{Z}^{2}+\lambda_{X}\gamma_{Z}\left(\lambda_{X}+\gamma_{Z}\right)-{\left(\lambda_{X}+\gamma_{Z}\right)}^{2}d. \end{array}$$

We shall consider the following three cases separately.

- $d<\lambda_{X}$
  In this case $g_{1}>0$ and consequently
  (14) $$\begin{array}{c} \lambda_{Q}=\frac{-b+b\sqrt{1-\frac{4ac}{b^{2}}}}{2a} \end{array}$$
  when *ℓ* is sufficiently large. Note that
  (15) $$\begin{array}{c} \sqrt{1-\frac{4ac}{b^{2}}}=1-\frac{2ac}{b^{2}}-\frac{2a^{2}c^{2}}{b^{4}}+O\left(\frac{1}{\ell^{3}}\right). \end{array}$$
  Substituting (15) into (14) gives
  (16) $$\begin{array}{c} \lambda_{Q}=-\frac{c}{b}-\frac{ac^{2}}{b^{3}}+O\left(\frac{1}{\ell^{2}}\right). \end{array}$$
  It is easy to show that
  (17) $$\begin{array}{c} -\frac{c}{b}=-\frac{h_{1}}{g_{1}}-\frac{g_{1}h_{2}-g_{2}h_{1}}{g_{1}^{2}\ell }+O\left(\frac{1}{\ell^{2}}\right), \end{array}$$
  (18) $$\begin{array}{c} -\frac{ac^{2}}{b^{3}}=-\frac{\left(\gamma_{X}-d\right)h_{1}^{2}}{g_{1}^{3}\ell }+O\left(\frac{1}{\ell^{2}}\right). \end{array}$$
  Combining (16), (17) and (18) yields
  $$\begin{array}{c} \lambda_{Q}=\eta_{1}+\frac{\eta_{2}}{\ell }+O\left(\frac{1}{\ell^{2}}\right), \end{array}$$
  where
  $$\begin{array}{c} \eta_{1}≜-\frac{h_{1}}{g_{1}},\\ \eta_{2}≜-\frac{g_{1}^{2}h_{2}-g_{1}g_{2}h_{1}+\left(\gamma_{X}-d\right)h_{1}^{2}}{g_{1}^{3}}. \end{array}$$
  Moreover, it can be verified via algebraic manipulations that
  $$\begin{array}{c} \eta_{1}=\frac{\left(\lambda_{X}+\gamma_{Z}\right)d-\lambda_{X}\gamma_{Z}}{\lambda_{X}-d},\\ \eta_{2}=-\frac{\lambda_{X}^{2}d^{2}}{{\left(\lambda_{X}-d\right)}^{3}}. \end{array}$$
  Now we write $\bar{r}\left(d\right)$ equivalently as
  (19) $$\begin{array}{c} \bar{r}\left(d\right)=\frac{1}{2}\log\frac{\ell \rho_{X}\gamma_{X}+\lambda_{X}+\gamma_{Z}+\lambda_{Q}}{\lambda_{X}+\gamma_{Z}+\lambda_{Q}}+\frac{\ell }{2}\log\frac{\lambda_{X}+\gamma_{Z}+\lambda_{Q}}{\lambda_{Q}}. \end{array}$$
  Note that
  (20) $$\begin{array}{cc} \frac{1}{2}\log\frac{\ell \rho_{X}\gamma_{X}+\lambda_{X}+\gamma_{Z}+\lambda_{Q}}{\lambda_{X}+\gamma_{Z}+\lambda_{Q}} & =\frac{1}{2}\log\ell +\frac{1}{2}\log\frac{\rho_{X}\gamma_{X}}{\lambda_{X}+\gamma_{Z}+\eta_{1}}+O\left(\frac{1}{\ell }\right)\\ & =\frac{1}{2}\log\ell +\frac{1}{2}\log\frac{\rho_{X}\gamma_{X}\left(\lambda_{X}-d\right)}{\lambda_{X}^{2}}+O\left(\frac{1}{\ell }\right) \end{array}$$
  and
  (21) $$\begin{array}{c} \frac{1}{2}\log\frac{\lambda_{X}+\gamma_{Z}+\lambda_{Q}}{\lambda_{Q}}\\ =\frac{1}{2}\log\frac{\lambda_{X}+\gamma_{Z}+\eta_{1}}{\eta_{1}}-\frac{\left(\lambda_{X}+\gamma_{Z}\right)\eta_{2}}{2\left(\lambda_{X}+\gamma_{Z}+\eta_{1}\right)\eta_{1}\ell }+O\left(\frac{1}{\ell^{2}}\right)\\ =\frac{1}{2}\log\frac{\lambda_{X}^{2}}{\left(\lambda_{X}+\gamma_{Z}\right)d-\lambda_{X}\gamma_{Z}}+\frac{\left(\lambda_{X}+\gamma_{Z}\right)d^{2}}{2\left(\lambda_{X}-d\right)\left(\left(\lambda_{X}+\gamma_{X}\right)d-\lambda_{X}\gamma_{Z}\right)\ell }+O\left(\frac{1}{\ell^{2}}\right). \end{array}$$
  Substituting (20) and (21) into (19) gives
  $$\begin{array}{cc} \bar{r}\left(d\right) & =\frac{\ell }{2}\log\frac{\lambda_{X}^{2}}{\left(\lambda_{X}+\gamma_{Z}\right)d-\lambda_{X}\gamma_{Z}}+\frac{1}{2}\log\ell +\frac{1}{2}\log\frac{\rho_{X}\gamma_{X}\left(\lambda_{X}-d\right)}{\lambda_{X}^{2}}\\ & \;+\frac{\left(\lambda_{X}+\gamma_{Z}\right)d^{2}}{2\left(\lambda_{X}-d\right)\left(\left(\lambda_{X}+\gamma_{X}\right)d-\lambda_{X}\gamma_{Z}\right)}+O\left(\frac{1}{\ell }\right). \end{array}$$
- $d=\lambda_{X}$
  In this case $g_{1}=0$ and consequently
  (22) $$\begin{array}{c} \lambda_{Q}=\frac{-g_{2}+\sqrt{g_{2}^{2}-4\left(\gamma_{X}-\lambda_{X}\right)\left(h_{1}\ell +h_{2}\right)}}{2(\gamma_{X}-\lambda_{X})}. \end{array}$$
  Note that
  (23) $$\begin{array}{c} \sqrt{g_{2}^{2}-4\left(\gamma_{X}-\lambda_{X}\right)\left(h_{1}\ell +h_{2}\right)}=\sqrt{-4\left(\gamma_{X}-\lambda_{X}\right)h_{1}\ell }+O\left(\frac{1}{\sqrt{\ell }}\right). \end{array}$$
  Substituting (23) into (22) gives
  $$\begin{array}{c} \lambda_{Q}=\mu_{1}\sqrt{\ell }+\mu_{2}+O\left(\frac{1}{\sqrt{\ell }}\right), \end{array}$$
  where
  $$\begin{array}{c} \mu_{1}≜\sqrt{-\frac{h_{1}}{\gamma_{X}-\lambda_{X}}},\\ \mu_{2}≜-\frac{g_{2}}{2(\gamma_{X}-\lambda_{X})}. \end{array}$$
  Moreover, it can be verified via algebraic manipulations that
  $$\begin{array}{c} \mu_{1}=\lambda_{X},\\ \mu_{2}=\frac{{\left(1-\rho_{X}\right)}^{2}\gamma_{X}-2\rho_{X}\gamma_{Z}}{2\rho_{X}}. \end{array}$$
  Now we proceed to derive an asymptotic expression of $\bar{r}\left(d\right)$. Note that
  (24) $$\begin{array}{cc} \frac{1}{2}\log\frac{\ell \rho_{X}\gamma_{X}+\lambda_{X}+\gamma_{Z}+\lambda_{Q}}{\lambda_{X}+\gamma_{Z}+\lambda_{Q}} & =\frac{1}{4}\log\ell +\frac{1}{2}\log\frac{\rho_{X}\gamma_{X}}{\mu_{1}}+O\left(\frac{1}{\sqrt{\ell }}\right)\\ & =\frac{1}{4}\log\ell +\frac{1}{2}\log\frac{\rho_{X}}{1-\rho_{X}}+O\left(\frac{1}{\sqrt{\ell }}\right) \end{array}$$
  and
  (25) $$\begin{array}{cc} \frac{1}{2}\log\frac{\lambda_{X}+\gamma_{Z}+\lambda_{Q}}{\lambda_{Q}} & =\frac{\lambda_{X}+\gamma_{Z}}{2\lambda_{Q}}-\frac{{\left(\lambda_{X}+\gamma_{Z}\right)}^{2}}{4\lambda_{Q}^{2}}+O\left(\frac{1}{\ell^{\frac{3}{2}}}\right)\\ & =\frac{\lambda_{X}+\gamma_{Z}}{2\mu_{1}\sqrt{\ell }}-\frac{\left(\lambda_{X}+\gamma_{Z}\right)\left(\lambda_{X}+\gamma_{Z}+2\mu_{2}\right)}{4\mu_{1}^{2}\ell }+O\left(\frac{1}{\ell^{\frac{3}{2}}}\right)\\ & =\frac{\lambda_{X}+\gamma_{Z}}{2\lambda_{X}\sqrt{\ell }}-\frac{\left(\lambda_{X}+\gamma_{Z}\right)\left(\lambda_{X}-\rho_{X}\gamma_{Z}\right)}{4\rho_{X}\lambda_{X}^{2}\ell }+O\left(\frac{1}{\ell^{\frac{3}{2}}}\right). \end{array}$$
  Substituting (24) and (25) into (19) gives
  $$\begin{array}{c} \bar{r}\left(\lambda_{X}\right)=\frac{\left(\lambda_{X}+\gamma_{Z}\right)\sqrt{\ell }}{2\lambda_{X}}+\frac{1}{4}\log\ell +\frac{1}{2}\log\frac{\rho_{X}}{1-\rho_{X}}-\frac{\left(\lambda_{X}+\gamma_{Z}\right)\left(\lambda_{X}-\rho_{X}\gamma_{Z}\right)}{4\rho_{X}\lambda_{X}^{2}}+O\left(\frac{1}{\sqrt{\ell }}\right). \end{array}$$
- $d>\lambda_{X}$
  In this case $g_{1}<0$ and consequently
  (26) $$\begin{array}{c} \lambda_{Q}=\frac{-b-b\sqrt{1-\frac{4ac}{b^{2}}}}{2a} \end{array}$$
  when *ℓ* is sufficiently large. Note that
  (27) $$\begin{array}{c} \sqrt{1-\frac{4ac}{b^{2}}}=1+O\left(\frac{1}{\ell }\right). \end{array}$$
  Substituting (27) into (26) gives
  (28) $$\begin{array}{c} \lambda_{Q}=-\frac{b}{a}+O\left(1\right). \end{array}$$
  It is easy to show that
  (29) $$\begin{array}{c} -\frac{b}{a}=\frac{\rho_{X}\gamma_{X}\left(d-\lambda_{X}\right)\ell }{\gamma_{X}-d}+O\left(1\right). \end{array}$$
  Combining (28) and (29) yields
  $$\begin{array}{c} \lambda_{Q}=\frac{\rho_{X}\gamma_{X}\left(d-\lambda_{X}\right)\ell }{\gamma_{X}-d}+O\left(1\right). \end{array}$$
  Now we proceed to derive an asymptotic expression of $\bar{r}\left(d\right)$. Note that
  (30) $$\begin{array}{c} \frac{1}{2}\log\frac{\ell \rho_{X}\gamma_{X}+\lambda_{X}+\gamma_{Z}+\lambda_{Q}}{\lambda_{X}+\gamma_{Z}+\lambda_{Q}}=\frac{1}{2}\log\frac{\rho_{X}\gamma_{X}}{d-\lambda_{X}}+O\left(\frac{1}{\ell }\right) \end{array}$$
  and
  (31) $$\begin{array}{cc} \frac{1}{2}\log\frac{\lambda_{X}+\gamma_{Z}+\lambda_{Q}}{\lambda_{Q}} & =\frac{\lambda_{X}+\gamma_{Z}}{2\lambda_{Q}}+O\left(\frac{1}{\ell^{2}}\right)\\ & =\frac{\left(\lambda_{X}+\gamma_{Z}\right)\left(\gamma_{X}-d\right)}{2\rho_{X}\gamma_{X}\left(d-\lambda_{X}\right)\ell }+O\left(\frac{1}{\ell^{2}}\right). \end{array}$$
  Substituting (30) and (31) into (19) gives
  $$\begin{array}{c} \bar{r}\left(d\right)=\frac{1}{2}\log\frac{\rho_{X}\gamma_{X}}{d-\lambda_{X}}+\frac{\left(\lambda_{X}+\gamma_{Z}\right)\left(\gamma_{X}-d\right)}{2\rho_{X}\gamma_{X}\left(d-\lambda_{X}\right)}+O\left(\frac{1}{\ell }\right). \end{array}$$
  This completes the proof of Theorem 2.

## 4. Conclusions

We have studied the problem of symmetric remote Gaussian source coding and made a systematic comparison of centralized encoding and distributed encoding in terms of the asymptotic rate-distortion performance. It is of great interest to extend our work by considering more general source and noise models.
